# Supplementary figures and images for: Characterization of the Nitrate Transporter gene family and functional identification of HvNRT2.1 in barley (Hordeum vulgare L.)
Source: PLoS One. 2020 Apr 23;15(4):e0232056. doi: 10.1371/journal.pone.0232056 (PMC7179922; doi:10.1371/journal.pone.0232056)

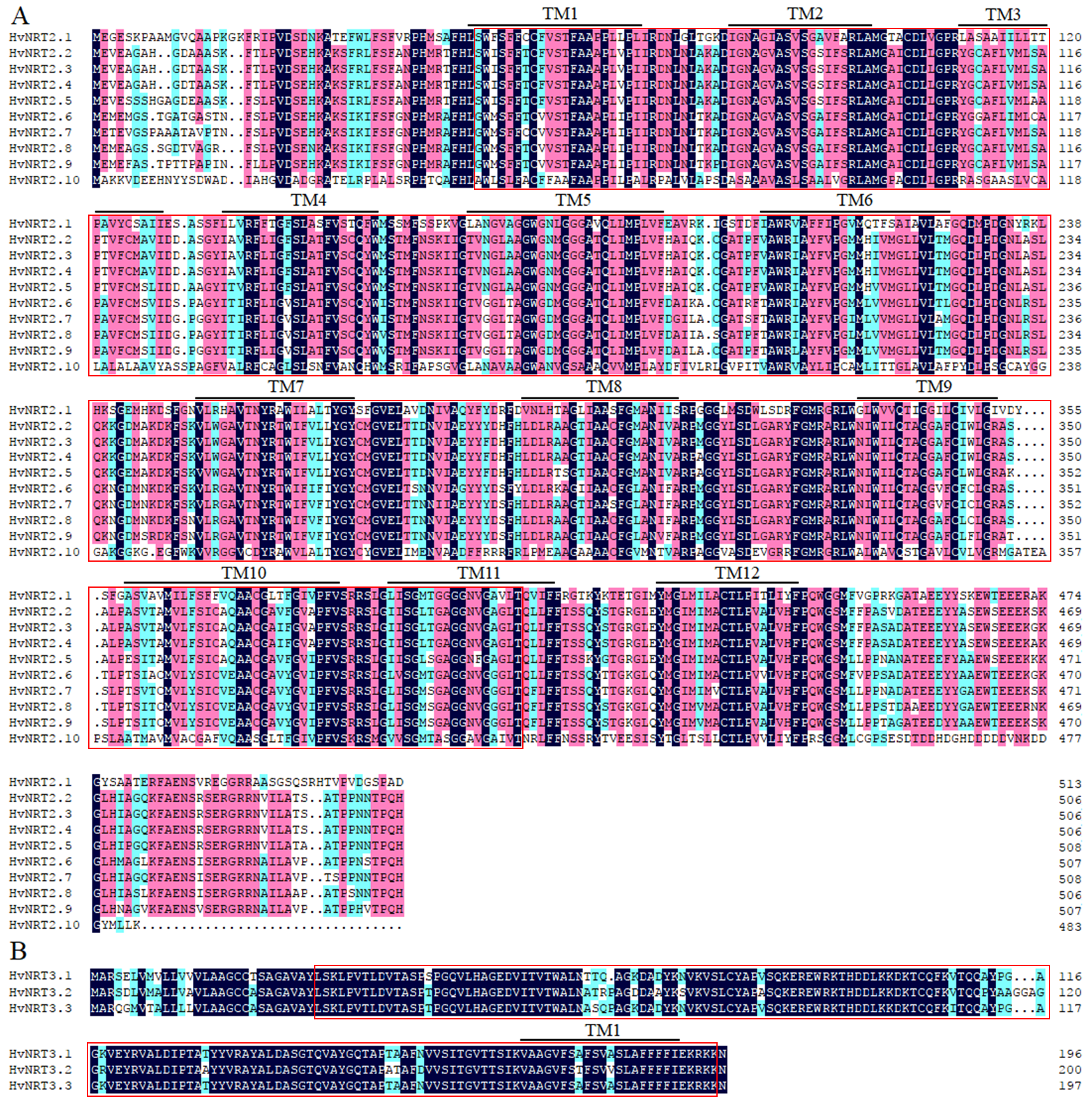

Supplement: S1 Fig — (TIF) [file pone.0232056.s001.tif]

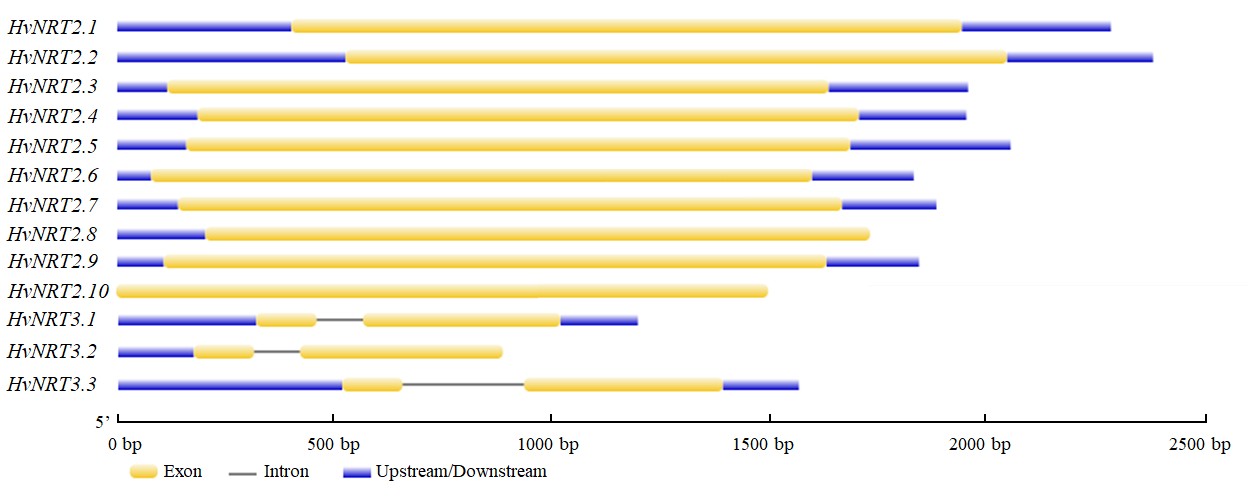

Supplement: S2 Fig — Amino acid sequence alignments of the HvNRT2 (A) and HvNRT3 (B) proteins. The conserved transmembrane sequence regions are indicated by the black lines above the sequences. The red frame indicates the MFS and NAR domains in the HvNRT2 and HvNRT3 proteins, respectively. (TIF) [file pone.0232056.s002.tif]

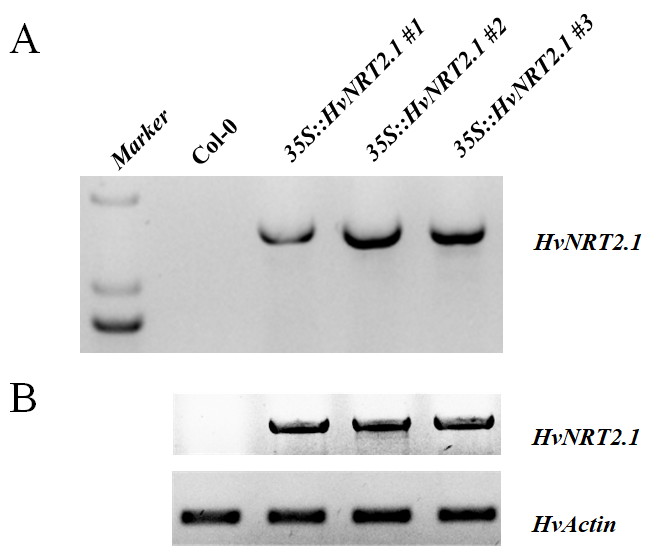

Supplement: S3 Fig — A, PCR analysis at the genomic level; B, RT-PCR analysis at the transcription level. (TIF) [file pone.0232056.s003.tif]
