# Supplementary material for: Characterization of the Nitrate Transporter gene family and functional identification of HvNRT2.1 in barley (Hordeum vulgare L.)
Source: PLoS One. 2020 Apr 23;15(4):e0232056. doi: 10.1371/journal.pone.0232056 (PMC7179922; doi:10.1371/journal.pone.0232056)
Supplement: Attachment — Submitted filename: Response to reviewers.docx [file pone.0232056.s006.docx]

**Responses to reviewer 1:**

Comment 1: A short introduction referring to the current understanding of individual HvNRT2/3 gene in cultivated barley is suggested.

Response 1: According to the reviewer’s suggestion, we have added the sentence in the revised manuscript. In barley, four *HvNRT2* (*HvNRT2.1*-*2.4*) and three HvNRT3 (*HvNAR2.1*-*2.3*) genes were isolated from the roots. The expression pattern of the *HvNRT*2 genes was also characterized under various nitrogen sources, and a highly specific interaction is suggested between *HvNRT2.1* and *HvNAR2.3* by using ^15^N-enriched nitrate uptake into Xenopus oocytes. However, until now, none of the *HvNRT2/3* gene family have been described at the barley genome level and their functions are still unclear.

Comment 2: Fig. 7 showing the increases of nitrate and total nitrogen content should be described in Results. This might be important in order to support the story of this study.

Response 2: We agree with this suggestion and have added the result in the “Morphological characterization of the HvNRT2.1 gene overexpression plants” paragraph as follows: In addition, seeds of overexpression plant accumulated significantly more nitrate and nitrogen, and increased by 47.2% and 41.4% compared with wide type in mature plants (Fig 7).

Comment 3: Use Arabidopsis thaliana or A. thaliana. Refer the variety “Col-0” in Materials.

Response 3: Thank the reviewer for this content. The *Arabidopsis thaliana* Columbia-0 (Col-0) ecotype was used as in materials. We have revised this in the new manuscript (see “Plant materials” part)

Comment 4: Fig. 4, statistical analysis is recommended (e.g. Tukey’s HSD).

Response: Thank the reviewer’s suggestion, statistical analysis of the differences between wide type (Col-0) and transgenic plants was performed by using Student’s *t*-test. We have added the result of Student’s *t*-test in Fig 6 and Fig 7.

Comment 5: The language editing is suggested.
Response: The statements have been corrected. We will be happy to edit the MS further base on helpful comments from reviewers.

**Responses to reviewer 2:**

**Major issues**

Comment 1: The English language needs professional proofreading as there are massive typos and grammar errors in the main document.
Response 1: The statements have been corrected. We really hope the flow and language level have been improved.

Comment 2: Why the *HvNRT2.1* but not other NRT transporters were specifically investigated in *Arabidopsis*?
Response 2: *HvNRT2.1* gene was the first to respond to low nitrogen with peaks at 1 h, which has high homology to *OsNRT2.3* (a gene derived from rice which has been contribute to improvement grain yield and NUE by 40%). Therefore, *HvNRT2.1* was specifically investigated in *Arabidopsis*.

Comment 3: Line 223, information missing in the sentence.

Response 3: Thank the reviewer for this content. The sentence was as follows: A total of 18 *NRT2* genes belonged to cluster Ⅰ, including five *NRT2* genes derived from dicotyledonous *Arabidopsis* and 13 *NRT2* genes from monocotyledonous plants (8, 2, and 3 genes from barley, rice, and maize, respectively). *AtNRT2.5* and *AtNRT2.7* represented clusters Ⅱ and Ⅲ, containing four and three genes, according to the phylogenetic tree, respectively (Fig 2A). We have revised this in the new manuscript.

Comment 4: Line 256, peaks at ??

Response 4: Thank the reviewer’s for this content. We have revised this as follows: The expression of *HvNRT3.3* displayed two peaks at 3 h and 24 h and responded more quickly to high rather than low nitrogen conditions.

Comment 5. Line 261, how much similar are they (% identity)?

Response 5: According to the reviewer’s suggestion. We analyzed the homology identity between HvNRT2.1 and OsNRT2.3. The detail information was as follow: The HvNRT2.1 protein had a similar amino acid length and showed 83.3% identity with *OsNRT2.3a*, but only 78.4% identity with *OsNRT2.3b* genes, indicating that *HvNRT2.1* plays an important role in long-distance nitrate transport, from the roots to shoots, with low nitrate supply levels in barley. We have added this in the new manuscript (see “Discussion” part).

Comment 6: Line 264, was Arabidopsis ecotype Col-0 used? Should not be in italic.

Response 6: Thank the reviewer for this content. The *Arabidopsis thaliana* Columbia-0 (Col-0) ecotype was used as in materials. We have revised this in the new manuscript (see “Plant materials” part)

Comment 7: Line 267, how much increments?

Response 7: According to the reviewer’s suggestion, the increments were calculated in the new revised manuscript. The detail information was as follows: 7.9%, 18.5%, and 23.3% increments in the length of siliques, yield per plant and grain number per siliques, respectively, were observed in the transgenic lines compared with Col-0 (Fig 6C, E, and F).

Comment 8. Line 278, two NRT3 from which species?

Response 8: Two NRT3 have been analyzed from rice, Arabidopsis and maize. Thus, we have revised this sentence in the manuscript as follows: a total of 4, 7, and 4 *NRT2* genes have been analyzed in rice, *Arabidopsis,* and maize, respectively, as well as two *NRT3* genes.

Comment 9. Line 282, what does “components” mean?

Response 9: For HATS activity in plants, NRT2/NRT3 is a two-component high-affinity nitrate transport system, each of them is part of high-affinity nitrate transport system and interact at the protein level.

Comment 10: Line 307-309, please specify the details in the changes.

Response 10: According to the reviewer’s suggestion, we have added the results in the “*HvNRT2*/*3* gene expression patterns at different developmental stages” and “*HvNRT2/3* gene expression analysis under low and high nitrogen levels” parts.

Comment 11: Line 313-318, state one or two functions in rice so that it can be more evidently related to barley.

Response 11: According to the reviewer’s suggestion, we have added the discussion in the revised manuscript as follows: In rice, a decreased expression of *OsNRT2.3a* is related to the accumulation of significantly higher levels of nitrate and total nitrogen in the root and lower levels in the shoot. However, enhancing the expression of *OsNRT2.3b* improves the growth, yield, and NUE in rice.

Comment 12. Line 335, the statement has been repeated a couple of times in the ms.

Response 12: Thank the reviewer for this content, we have deleted the repeat sentence about this in the revised manuscript.

Comment 13. Line 338, increase of biomass and plant height in Arabidopsis or ?? If so could be rephrased as “In the present study, overexpression of HvNRT2.1 led to high nitrate and nitrogen content, increased biomass and plant height and improved the yield-related traits in Arabidopsis indicating that….

Response 13: Thank the reviewer for this content. We check the result, and adjust the discussion in the new revised manuscript. The detail information as follows: In the present study, the overexpression of *HvNRT2.1* led to high nitrate and nitrogen contents and improved the yield-related traits of *Arabidopsis*. However, both a biomass and plant height increase were not observed in the overexpression plants, indicating that the *HvNRT2.1* gene plays a specific role in nitrate and total nitrogen accumulation in plants.

Comment 14. The authors should provide either qualitative or quantitive information regarding the *HvNRT2.1* transcript level in transgenic *Arabidopsis* lines.

Response 14: According to the reviewer’s suggestion. We have added this information in S3 Fig in the reviewed manuscript (see “Morphological characterization of the *HvNRT2.1* overexpression plants” part). The detail information as follows: In the transgenic lines, *HvNRT2.1* was introduced into the genomic DNA and was expressed at the transcription level in three independent overexpression plants

Comment 15: HvNRT2.1 expression in barley responded to different N levels so why not investigate the growth performance of transgenic Arabidopsis lines under N treatments?

Response 15: We agree with this suggestion. We have plant wide type and transgenic Arabidopsis lines on the MS (without NH4NO_3_) with different KNO_3_ level, unfortunately, all plants cannot survive. Thus, the growth performance of wide type and transgenic lines was observed on the normal conditions.

**Comment Minor issues**

Comment 1: Line 20, rectify the grammar issue

Response 1: Thank the reviewer for this comment. We have corrected the sentence as follow: Nitrogen use efficiency (NUE) is the efficiency with which plants acquire and use nitrogen.

Comment 2. Line 23, in barley “nitrate transport”

Response 2: Thank the reviewer for this content. We have added the word “nitrate transport” in the new manuscript.

Comment 3: Line 25, “chromosomes”

Response 3: Thank the reviewer for this comment. We have replaced the “chromosome” with “chromosomes”.

Comment 4: Line 29, replace “notably” with “furthermore”

Response 4: Thank the reviewer for this comment. We have replaced the “notably” with “furthermore”.

Comment 5: Line 55, should be “they are” but not “it is”

Response 5: Thank the reviewer for this comment. We have replaced the “it is” with “they are”.

Comment 6: Line 61, under low N condition?

Response 6: Nitrate supply was 0.5 mM in the reference (Tang et al., 2012), which was consider as low nitrate supply.

Comment 7: Line 86-89, confusing with the experimental layout. Indeed, how many plants were sampled and used for RNA extraction?

Response 7: According to the reviewer’s suggestion. We have revised this sentence as follow: At 0 h, 1 h, 3 h, 6 h, 12 h, 24 h, 48 h and 72 h after treatment, total roots were harvested from ten plants and immediately frozen in liquid nitrogen for RNA extraction with three biological replicates at each time point.

Comment 8: Line 137, “0.5 cm” would be better.

Response 8: Thank the reviewer for this comment. We have replaced the “mm” with “cm”.

Comment 9: Line 224, according “to”

Response 9: Thank the reviewer for this comment. We have added this word in the revised manuscript.

Comment 10. Line 228, “clustered”

Response 10: Thank the reviewer for this comment. We have a replaced the “cluster” with “clustered”

Comment 11: Line 320, “encode”

Response 11: Thank the reviewer for this comment. The word “encoding” has been replace by “encode”.
