# Supplementary material for: Characterization of the Nitrate Transporter gene family and functional identification of HvNRT2.1 in barley (Hordeum vulgare L.)
Source: PLoS One. 2020 Apr 23;15(4):e0232056. doi: 10.1371/journal.pone.0232056 (PMC7179922; doi:10.1371/journal.pone.0232056)
Supplement: Attachment — Submitted filename: Response to reviewers.doc [file pone.0232056.s007.doc]

**Responses to reviewer 2**

Comment 1: The revised manuscript has been improved and I have the following comments in regard to typos or grammar errors. In addition, I suggest the authors adding the gene accession numbers or gene IDs such as HORVU.. as the last section of Methods, for a better understanding and traceback with other researches.

Response 1: Thank the reviewer for this comment. We have listed the gene IDs, chromosome position, coding sequence length, amino acid length, mass and *pI* in the Table 1 in the revised manuscript.

Comment 2: Line 37: Replace “serious” with “severe/intense”

Response 2: Thank the reviewer for this comment. We have replaced the “serious” with “severe”.

Comment 3: Line 48: Replace “shows” with “functions as”

Response 3: Thank the reviewer for this comment. We have replaced the “shows” with “functions as”.

Comment 4: Line 53: Can be rephrased as “There is a lesser number of NRT2 genes than NRT1”.

Response 4: We agreed with the reviewer’s suggestion, and rephrased as “There is a lesser number of NRT2 genes than NRT1” in the revised manuscript.

Comment 5: Line 177: “herbicide treatment”

Response 5: We agreed with the reviewer’s suggestion. we have added the word “treatment” after “herbicide”

Comment 6: Line 227: Check the 3 distinct clusters

Response 6: Thank the reviewer for this comment. The words“Ⅰ, Ⅱ, and Ⅲ”have not displayed properly in the PDF version. The sentence as follows: According to the phylogenetic tree of the NRT2 and NRT3 proteins in barley, rice, maize, and *Arabidopsis*, the NRT2 proteins could clearly be divided into three distinct clusters (Ⅰ, Ⅱ, and Ⅲ).

Comment 7: Line 230: It has to be cluster II and III?

Response 7: Thank the reviewer for this comment. The words“Ⅱ, Ⅲ”have not displayed properly in the PDF version. The sentence as follows: AtNRT2.5 and AtNRT2.7 represented clusters Ⅱ and Ⅲ, containing four and three genes, according to the phylogenetic tree, respectively.

Comment 8: Line 330: Replace “with” with “under”

Response 8: We agreed with the reviewer’s suggestion. We have replaced the “with” with “under”.
